# Poverty at Older Ages: Insights From a Pilot Principal Poverty Measure

**DOI:** 10.1093/geroni/igaf122.1517

**Published:** 2025-12-31

**Authors:** Rosemary Hyson, Sanders Korenman

**Affiliations:** Baruch College, CUNY, New York City, New York, United States; Baruch College, CUNY, New York City, New York, United States

## Abstract

This paper estimates poverty rates and impacts of government benefits on poverty according to a pilot Principal Poverty Measure (PPM) for 2020-2023 using March CPS data. The PPM is a revision of the Census Bureau’s Supplemental Poverty Measure (SPM) and was recommended by a 2023 NAS report. Our pilot PPM poverty line uses policy-based needs standards following an approach developed for the Health Inclusive Poverty Measure (Korenman and Remler 2016): a benchmark silver plan from ACA Health Insurance Marketplaces or Medicare coverage, Fair Market Rents from HUD, and dietary needs in USDA’s Thrifty Food Plans. PPM resources include cash income, in-kind benefits including health insurance, and, for homeowners, an implicit net resource flow from their home: avoided rental payments minus homeowner costs. Compared to the SPM, PPM poverty rates are lower among homeowners and those with health insurance benefits. Accordingly, the PPM estimates greater differences in poverty between children and older persons. PPM poverty gap analyses reveal that, together, social insurance, means-tested transfers and tax credits close about 96% of the pre-transfer PPM poverty gap for persons 65+, while substantial gaps remain for children. The PPM also finds large anti-poverty impacts of rental assistance: among older people who receive it, rental assistance reduces poverty by more than 50 percentage points according to the PPM, but only 34 percentage points according to the SPM. We will also project impacts on poverty of changes in federal healthcare, housing and food programs currently under discussion.

